# Exogenous Expression of an Alternative Splicing Variant of Myostatin Prompts Leg Muscle Fiber Hyperplasia in Japanese Quail

**DOI:** 10.3390/ijms20184617

**Published:** 2019-09-18

**Authors:** Paula Renee Chen, Yeunsu Suh, Sangsu Shin, Rachel Marie Woodfint, Seongsoo Hwang, Kichoon Lee

**Affiliations:** 1 Department of Animal Science, The Ohio State University, Columbus, OH 43210, USA; prcn78@mail.missouri.edu (P.R.C.); suh.83@osu.edu (Y.S.); woodfint.1@osu.edu (R.M.W.); 2 Department of Animal Biotechnology, Kyungpook National University, Sangju 37224, Korea; sss@knu.ac.kr; 3 Animal Biotechnology Division, National Institute of Animal Science, RDA, Wanju-gun, Jeonbuk 55365, Korea; hwangss@korea.kr; 4 The Ohio State University Interdisciplinary Human Nutrition Program, The Ohio State University, Columbus, OH 43210, USA

**Keywords:** myostatin, skeletal muscle, alternative splicing, transgenic, avian

## Abstract

Myostatin (MSTN) negatively regulates muscle growth and development through inhibiting myoblast proliferation and differentiation. Five alternative splicing isoforms of MSTN (MSTN-A to MSTN-E) have been discovered in domestic avian species. MSTN-A has high expression in skeletal muscle and encodes the full-length peptide with anti-myogenic activity. Another isoform, MSTN-B, is also highly expressed in skeletal muscle and encodes a truncated peptide that has pro-myogenic capabilities in vitro, which include promoting the proliferation and differentiation of quail muscle precursor cells. The objective of this study was to investigate overexpression of MSTN-B in vivo by using two independent lines of transgenic Japanese quail with expression directed in the skeletal muscle. Unexpectedly, the chicken skeletal muscle alpha actin 1 (cACTA1) promoter resulted in restricted exogenous MSTN-B protein expression to certain skeletal muscles, such as the gastrocnemius and tibialis anterior, but not the pectoralis major muscle. Gastrocnemius weight as a percentage of body weight in transgenic quail was increased compared to non-transgenic quail at posthatch day 21 (D21) and posthatch D42. An increase in the size of the gastrocnemius in transgenic quail was attributed to an increase in fiber number but not fiber cross-sectional area (CSA). During embryonic development, paired box 7 (PAX7) expression was prolonged in the transgenic embryos, but other myogenic regulatory factors (MRFs) were unchanged after MSTN-B overexpression. Taken together, these data provide novel insights into the regulation of skeletal muscle development by alternative splicing mechanisms in avians.

## 1. Introduction

Poultry consumption in the United States has steadily increased throughout the years; therefore, discovering ways of improving muscle accretion is warranted. The weight of a muscle is ultimately a result of proliferation of myoblasts during embryonic stages, hypertrophy of muscle fibers through protein synthesis, and donation of nuclei by satellite cells after hatch [1]. Genetic selection of domestic poultry species has greatly enhanced muscle growth [2], but further investigation of the cellular and molecular mechanisms involved in skeletal muscle development is required.

Myostatin (MSTN) is a member of the transforming growth factor-β (TGF-β) superfamily and is an anti-myogenic factor in humans and animals. After translation, the resulting peptide, known as pro-MSTN, undergoes three proteolytic processing events. Initially, a signal peptidase removes the N-terminal signal sequence. Then, pro-MSTN dimerizes near the C-terminus and is systematically cleaved at the RXXR site by a calcium-dependent serine protease, known as furin, to generate the N-terminal propeptides and C-terminal receptor-binding domain [3]. However, the latent MSTN complex forms as the propeptides noncovalently bind the C-terminal region via a critical peptide sequence, which prevents MSTN from binding to its target receptor. Members of the bone morphogenetic protein-1/tolloid (BMP-1/TLD) family are metalloproteinases that can cleave the propeptides from the latent complex and release mature MSTN, which can bind activin receptor type 2B (ACVR2B) to induce the appropriate signaling cascade [4].

Determining methods of inhibiting MSTN processing and signaling is of great interest for poultry and livestock producers as well as the medical field for treating muscle-wasting diseases and muscle weakness [5]. Natural mutations in the *MSTN* gene that disrupt the active C-terminal region have been shown to markedly increase muscle mass in several species, including cattle, dogs, and even humans [6,7,8]. Follistatin, a dominant-negative form of ACVR2B, and the MSTN propeptide have been investigated as applicable inhibitors of MSTN [9,10,11]. While all three resulted in increased muscle mass by hyperplasia and hypertrophy, follistatin had the most profound effect, and directly binds to MSTN to prevent binding to ACVR2B [12]. Antibodies have been developed that bind to MSTN or act as competitive inhibitors by binding to ACVR2B, and these antibodies have also produced increases in muscle mass in mice and chickens [13,14,15,16]. Moreover, knockdown of ACVR2B by shRNAs increased body and muscle weights in chickens, confirming the importance of this receptor in the signaling cascade [17]. Multiple MSTN isoforms produced by alternative splicing have been revealed in avians (MSTN-A to MSTN-E) [18]. The full-length MSTN peptide, encoded by MSTN-A, and another isoform, MSTN-B, which encodes a truncated peptide devoid of the active C-terminal region, are highly expressed in skeletal muscle. In vitro analysis of MSTN-B demonstrated that this isoform promoted proliferation and differentiation of quail myogenic cells and binds to MSTN-A to inhibit proteolytic processing and the release of mature MSTN [19]. The purpose of the present study was to generate a novel transgenic quail model overexpressing MSTN-B in skeletal muscle and determine the effects of this isoform in vivo. Characterization of the potential pro-myogenic effects of MSTN-B will lead to further understanding of protein regulation by alternative splicing mechanisms.

## 2. Results

### 2.1. Production of MSTN-B Transgenic Quail

**Figure 1:**
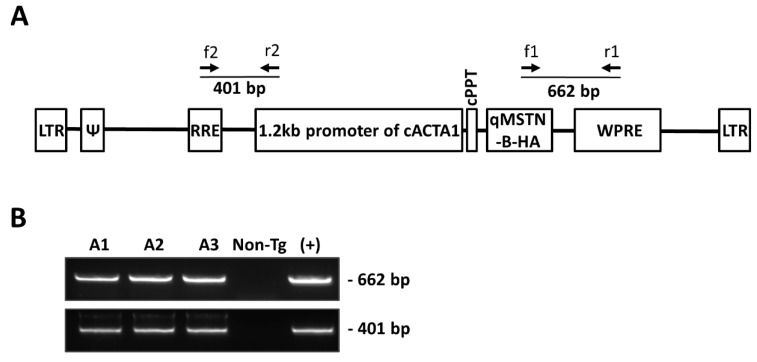
Diagram of lentiviral vector used for generating myostatin-B (*MSTN-B*) transgenic quail and confirmation of transgene insertion by PCR. (**A**) The vector contained the 1.2-kb promoter of chicken skeletal muscle alpha actin 1 (cACTA1) and quail *MSTN-B* coding sequences with a hemagglutinin (HA) tag. The primers for detecting the transgene are presented as f1 (HAtag-F) and f2 (RRE-F) for forward primers and r1 (WPRE-R) and r2 (cACTA1-R) for reverse primers. (**B**) All cACTA1-qMSTN-B transgenic quail were selected by PCR using two primer sets, f1 + r1 (662 bp) and f2 + r2 (401 bp). The positive control (+) is the lentiviral vector.

A lentiviral vector containing the 1.2-kb promoter of chicken skeletal muscle alpha actin 1 (cACTA1) and quail *MSTN-B* with a hemagglutinin (HA) tag was constructed to express exogenous MSTN-B strictly in the skeletal muscle (Figure 1A). A total of 93 wild-type quail eggs were injected with the recombinant lentivirus; fourteen founder chicks hatched, and 11 grew to sexual maturity to be crossed with wild-type quail. After screening the G1 offspring, three lines were confirmed by PCR (A1–A3) (Figure 1B, Table 1). 

**Figure 2:**
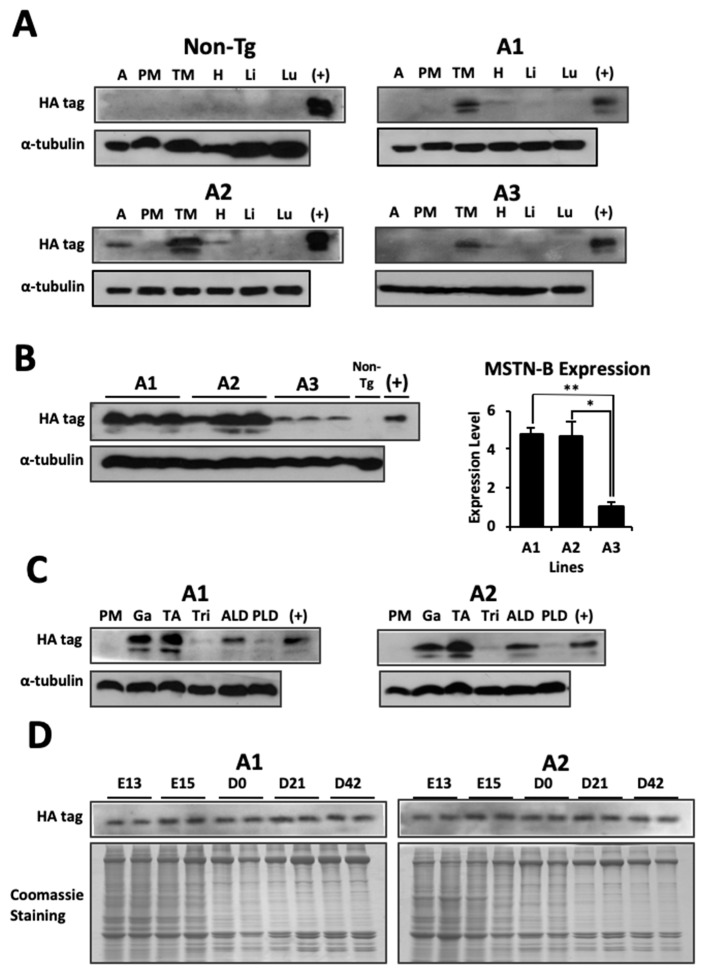
Representative western blots of MSTN-B protein expression in non-transgenic (Non-Tg) and transgenic quail lines. (**A**) Tissue distribution, including adipose tissue (A), pectoralis major muscle (PM), thigh muscle (TM), heart (H), liver (Li), and lung (Lu), demonstrating the specificity of MSTN-B protein with the HA tag. (**B**) Three random six-week-old transgenic quail from each line were used to demonstrate the expression of MSTN-B with an HA tag in thigh muscle. One non-transgenic quail was used as a negative control. Values of densitometry analysis are represented as means ± SEM (*n* = 3). * and ** indicates significance levels of *p* < 0.05 and *p* < 0.01, respectively. (**C**) A1 and A2 were selected for further analyses. Muscle distributions, including pectoralis major muscle (PM), gastrocnemius (Ga), tibialis anterior (TA), triceps brachii (Tri), anterior latissimus dorsi (ALD), and posterior latissimus dorsi (PLD), were performed to examine MSTN-B expression in different skeletal muscles. The positive control (+) was 293FT cells transfected with the lentiviral vector, and α-tubulin expression was used as a reference. (**D**) MSTN-B expression from transgene in embryos or quail from A1 and A2 across time points (*n* = 2) with Coomassie staining as a reference.

**Figure 3:**
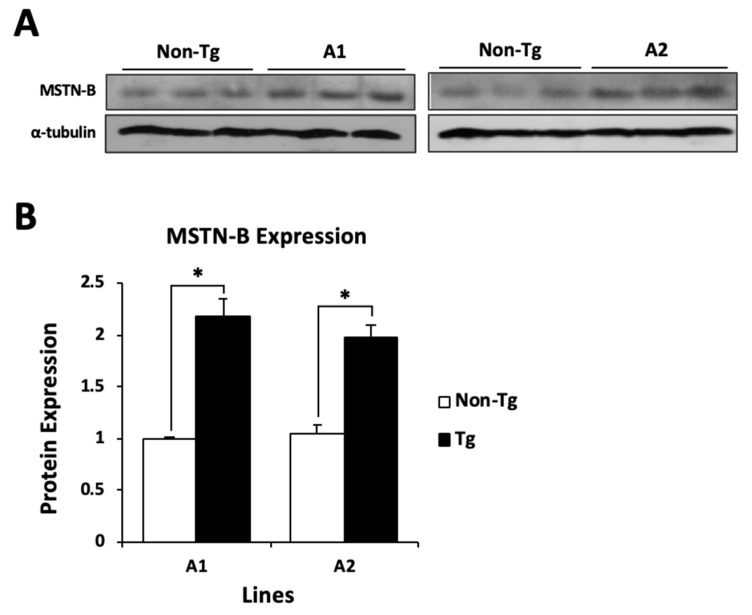
Three six-week-old non-transgenic (Non-Tg) and transgenic (Tg) quail from A1 and A2 were randomly selected to demonstrate (**A**) total (endogenous and exogenous) MSTN-B expression in the gastrocnemius, and α-tubulin expression was used as a reference. (**B**) Densitometry analysis for MSTN-B expression. * indicates a significance level of *p* < 0.05.

The expression of MSTN-B in skeletal muscle was confirmed by western blot analysis by using a mouse HA tag antibody. Unexpectedly, lines A1–A3 expressed MSTN-B protein in the thigh muscle, but not in the pectoralis major muscle. In addition, the three lines had a minor expression of MSTN-B in the heart, and A2 expressed MSTN-B in adipose tissue (Figure 2A). As shown by Figure 2B and after densitometry analysis, lines A1 and A2 expressed approximately 4.8 and 4.7-fold more MSTN-B protein in the thigh muscle than A3, respectively; therefore, these two lines were selected for further analyses. From the muscles that were tested for MSTN-B expression, the gastrocnemius, tibialis anterior, and anterior latissimus dorsi were the only ones to demonstrate strong expression in both lines. The triceps brachii and posterior latissimus dorsi had minor expression, and the pectoralis major muscle did not exhibit presence of qMSTN-B (Figure 2C). Expression of MSTN-B was detected in transgenic embryos and quail from A1 and A2 at all developmental time points collected (Figure 2D). When incubated with a MSTN antibody to detect endogenous and exogenous MSTN-B, A1 transgenic quail had 2.2-fold more MSTN-B expression in the gastrocnemius, and A2 transgenic quail had 2.0-fold more MSTN-B expression in the gastrocnemius compared to non-transgenic quail from their respective lines (Figure 3A,B).

### 2.2. MSTN-B Overexpression and Muscle Development

**Figure 4:**
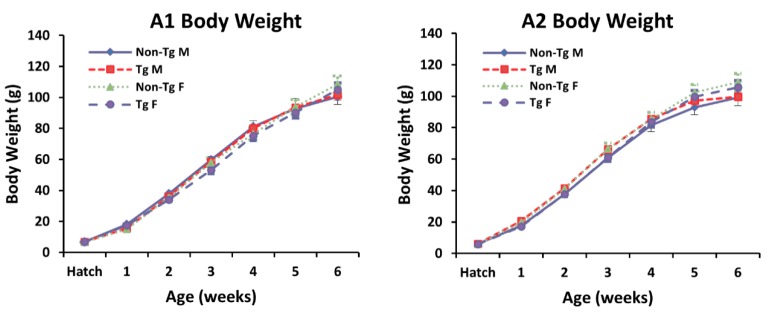
Growth curves for quail from A1 and A2 over a six-week period. Body weight (g) was measured each week for quail from A1 and A2 (*n* = 5 per group). Non-transgenic and transgenic males (M) and females (F) were weighed from each line.

During the six-week growth period, body weights did not differ between non-transgenic and transgenic quail (Figure 4). As shown in Table 2, pectoralis major muscle weight as a percentage of body weight was not different between transgenic and non-transgenic quail at D21 or D42. In addition, heart weight as a percentage of body weight was not different between the groups. Comparison of the right gastrocnemius weight as a percentage of body weight revealed that transgenic males had a 9.4% increase in gastrocnemius weight at D21 and an 8.4% increase at D42 compared to non-transgenic males. Transgenic females had an 8.9% increase in gastrocnemius weight at D21 and a 14.6% increase in gastrocnemius weight at D42 compared to non-transgenic females (Table 2). These results indicate that MSTN-B overexpression can create modest increases in muscle weights where the exogenous protein is localized.

### 2.3. Histological Analysis

To determine the effects of MSTN-B overexpression at the cellular level, a portion of the pectoralis major and left gastrocnemius were sectioned and stained with hematoxylin and eosin. As shown in Table 3, fiber cross-sectional area (CSA) of the pectoralis major muscle was not different at D21 or D42 between any groups, which is possibly because the MSTN-B protein was not present. Interestingly, fiber CSA of the left gastrocnemius was not different at D21 or D42 between any groups, eliminating fiber hypertrophy as a contributor to increased gastrocnemius weight in transgenic quail. Total fiber number was increased in all male and female transgenic groups compared to non-transgenic quail. Transgenic males had approximately 9.2% more muscle fibers at D21 and 10.7% more fibers at D42 than non-transgenic males. Transgenic females had 10.3% more muscle fibers at D21 and 11.1% more fibers at D42 than non-transgenic females. Thus, exogenous MSTN-B protein in the gastrocnemius resulted in muscle fiber hyperplasia but not hypertrophy. 

### 2.4. Expression of Myogenic Regulatory Factors

**Figure 5:**
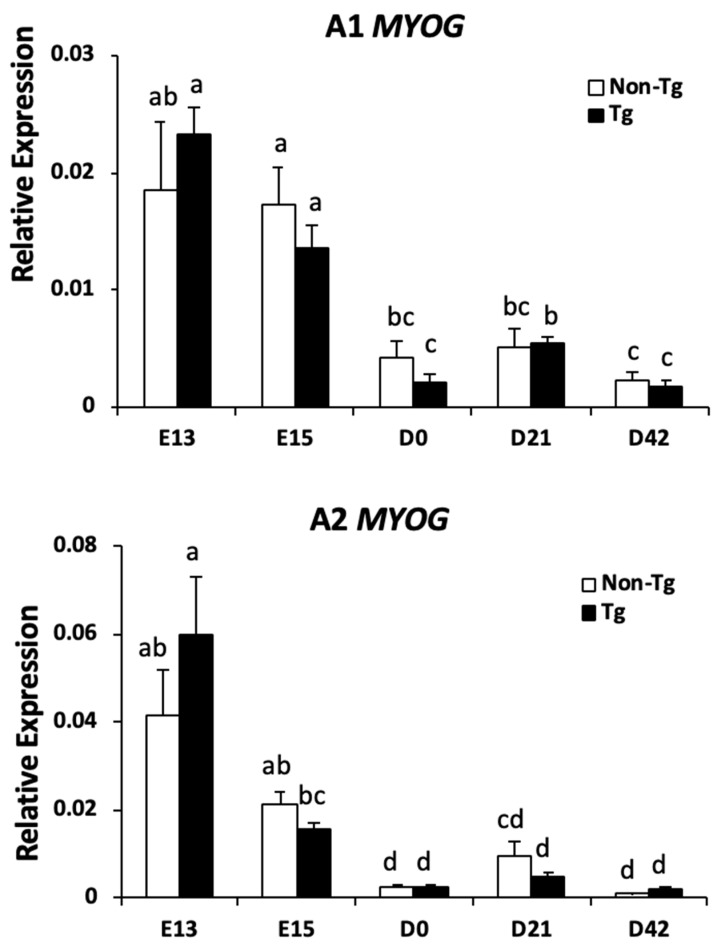
Transcript abundance of myogenin (*MYOG*) was measured by qPCR and normalized to *RPS13* for non-transgenic and transgenic embryos or quail at each time point from A1 and A2 (*n* = 5 per group per time point). Bars represent means ± SEM. Bars that have different superscript letters (^a–d^) are significantly different from each other (*p* < 0.05).

**Figure 6:**
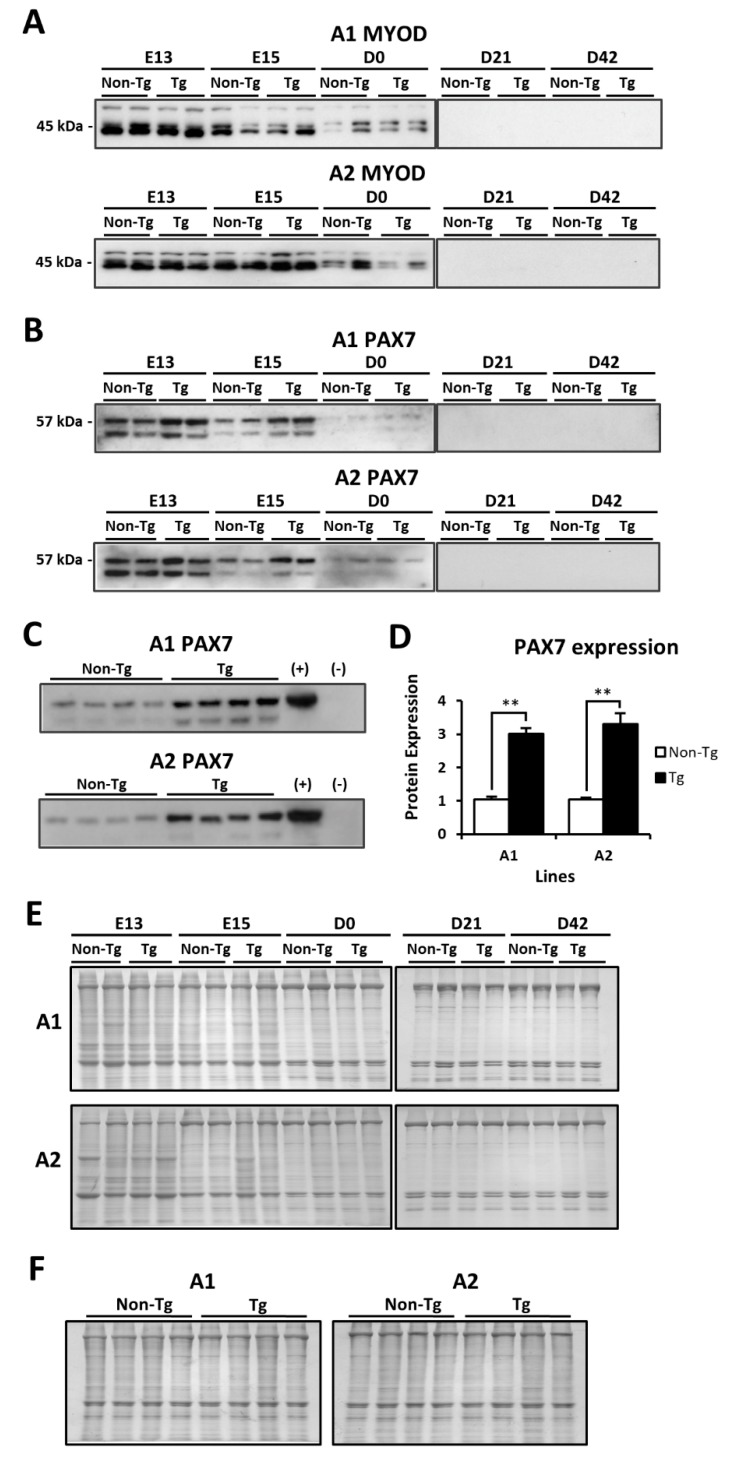
Time point comparison of (**A**) MYOD expression and (**B**) PAX7 expression between non-transgenic and transgenic embryos or quail from A1 and A2 (*n* = 2 per group per time point). (**C**) Analysis of PAX7 expression for A1 and A2 non-transgenic and transgenic embryos at E15 (*n* = 4 per group). HeLa cells overexpressing PAX7 were used as the positive control (+), and untransfected HeLa cells were used as the negative control (–). (**D**) Densitometry analysis for PAX7 at E15. ** indicates a significance level of *p* < 0.01. (**E**) Coomassie staining for A1 and A2 at each time point (*n* = 2 per group per time point) and (**F**) at E15 (*n* = 4 per group).

Quantitative real-time PCR analysis for myogenin (*MYOG*) revealed no differences in transcript abundance at any time point between non-transgenic and transgenic quail in either A1 or A2 (Figure 5). In addition, protein expression of myoblast determination protein 1 (MYOD) was not different between non-transgenic and transgenic quail from A1 or A2 at any time point, and expression began decreasing around the time of hatch and was undetectable by D21 (Figure 6A). Paired box 7 (PAX7) expression was prolonged in both A1 and A2 transgenic embryos until E15, whereas PAX7 expression in non-transgenic embryos was low at E15. Moreover, PAX7 expression decreased as development progressed and was not detectable by D21 in either non-transgenic or transgenic quail (Figure 6B). Further analysis of PAX7 expression at E15 revealed that A1 transgenic embryos had 3.0-fold greater expression, and A2 transgenic embryos had 3.3-fold greater expression than their respective non-transgenic counterparts (*p* < 0.01; Figure 6C,D), confirming prolonged expression of PAX7 during embryonic development. 

## 3. Discussion

Of the five MSTN isoforms produced by alternative splicing in avians, two isoforms, MSTN-A and MSTN-B, are highly expressed in skeletal muscle [18,19]. MSTN-A encodes the full-length peptide, which is a strong negative regulator of myoblast proliferation and differentiation [20]. Although in vitro studies revealed that MSTN-B, which encodes a truncated peptide containing a portion of the MSTN propeptide, enhanced proliferation and differentiation when overexpressed in quail muscle precursor cells [19], the pro-myogenic role of MSTN-B has not been studied in vivo. Japanese quail are emerging as a preferred animal model due to their small size, short period to sexual maturity, and high chromosomal homology with chickens [21]. In the present study, transgenic Japanese quail lines overexpressing quail *MSTN-B* under the control of the chicken skeletal muscle alpha actin 1 (cACTA1) promoter were generated to investigate the function of this isoform in vivo. A previous report by Petropoulos et al. demonstrated that the cACTA1 promoter was able to drive skeletal muscle-specific expression of chloramphenicol acetyltransferase in mice [22]. Unexpectedly, in the present study, the cACTA1 promoter was able to drive expression of the *MSTN-B* transgene in leg muscle but not the pectoralis major muscle. In addition, all lines had minor expression of MSTN-B in the heart, and quail from A2 had expression in the adipose tissue, which was likely due to random integration of the transgene into the genome. 

Although the exact cause of exogenous MSTN-B expression being mainly localized within the leg muscles is still unknown, the cACTA1 promoter could have possibly driven expression in only type I muscle fibers. Domestic poultry engage in very short bursts of flight; therefore, the pectoralis major muscle is almost exclusively composed of type II fibers to rapidly mobilize glycogen for this activity. Meanwhile, muscles in the legs contain heterogeneous populations of type I and type II fibers to increase endurance capabilities [23]. In the current study, the muscle distribution analysis of transgenic quail demonstrated high expression of exogenous MSTN-B in the leg muscles, gastrocnemius and tibialis anterior, as well as the anterior latissimus dorsi. Minor expression was detected in the triceps brachii and posterior latissimus dorsi, while the pectoralis major muscle did not have any expression. The anterior latissimus dorsi is defined as a tonic muscle and has a greater population of type I fibers, whereas the posterior latissimus dorsi is a fast-twitch muscle, and is mostly composed of type II fibers [24]. Additionally, the avian triceps brachii has a higher ratio of type II fibers to type I fibers [25].

Mutations within the skeletal muscle alpha actin gene have been linked to nemaline myopathy and congenital fiber type disproportion in humans. Analysis of muscle biopsies from patients with nemaline myopathy revealed a predominance of type I fibers, but many of the fibers were atrophied [26]. Three distinct missense mutations within the skeletal muscle alpha actin gene were linked with congenital fiber type disproportion. Interestingly, these mutations caused type I fiber hypotrophy, but type II fibers were unaffected [27]. Since skeletal muscle alpha actin is expressed in all skeletal muscle fibers, these mutations may have specific interactions with type I fiber pathways. Therefore, this 1.2-kb cACTA1 promoter may have directed expression of the MSTN-B transgene predominantly in type I fibers, but further investigation is required.

MSTN-B in avians contains the first 129 amino acids of the full-length MSTN sequence, which spans the beginning of the MSTN propeptide [19]. During the processing of MSTN, the N-terminal MSTN propeptides are cleaved from dimerized pro-MSTN by furin protease [3]. In addition, processing of another pro-MSTN dimer can be inhibited by the binding of two propeptide molecules [9]. Specific amino acid residues close to the amino terminus of the propeptide are necessary for the inhibitory activity. Using a bacterial expression system, 15 truncated versions of the MSTN propeptide were generated, and residues 42–115 were determined as being essential for MSTN inhibition [9]. Furthermore, the region containing residues 45–100 of the MSTN propeptide in flatfish had inhibitory activity with the same potency as the full propeptide sequence [28]. Therefore, the residues that are required for binding and inhibiting pro-MSTN processing are expected to be included in the MSTN-B peptide. This suggests that MSTN-B is another version of a short peptide that is derived from the MSTN propeptide but still has MSTN inhibitory capacity. 

During the six-week growth period, no difference in body weight was observed between non-transgenic and transgenic quail in either line. For each experimental quail, the pectoralis major muscle, right gastrocnemius, and heart were excised and weighed. Both male and female transgenic birds had right gastrocnemius muscles that were greater percentages of the body weight than their non-transgenic counterparts at D21 and D42, while the pectoralis major muscle weights and heart weights as percentages of body weight were not different. MSTN signals in paracrine and endocrine manners; however, paracrine signaling has more of an impact on the surrounding muscles [28]. In addition, broiler chickens with high feed efficiency have demonstrated decreased expression of MSTN signaling pathway genes, which may contribute to increases in muscle mass [29]. Since MSTN-B expression was localized to specific muscles, exogenous MSTN-B in the gastrocnemius may have been able to inhibit endogenous MSTN-A to result in increased size. 

Muscle fiber hyperplasia can increase muscle weight with or without fiber hypertrophy. Mice that did not have MSTN production in posteriorly-located muscles, the quadriceps and gastrocnemius, were generated by using the Cre-lox recombination system [28]. Posteriorly-located muscles had evident increases in weight compared to anteriorly-located muscles that produced MSTN. Interestingly, majority of the increases in posteriorly-located muscle weights were due to fiber hyperplasia, while fiber hypertrophy contributed to a lesser extent. The fiber number within the gastrocnemius of transgenic quail from both lines was increased compared to non-transgenic quail, while the fiber cross-sectional area (CSA) was not different. This indicates that proliferation of myogenic precursor cells was more active in transgenic quail, and the increase in gastrocnemius weight was likely due to muscle fiber hyperplasia. 

Temporal expression of myogenic regulatory factors (MRFs) impacts the ultimate weight of a muscle by regulating the proliferation of myogenic precursors as well as differentiation into fully functional muscle fibers. Paired box 7 (PAX7) is a transcription factor that is involved in developmental myogenesis and adult muscle regeneration. Within the mouse embryo, Pax7 promotes myogenic precursor cell proliferation and maintains the expression of other MRFs to enable differentiation to occur [30]. Similarly, quiescent satellite cell populations in adult skeletal muscle express Pax7. Once activated, satellite cells coexpress Pax7 with myogenic differentiation 1 (MyoD) to allow for proliferation followed by the downregulation of Pax7 and initiation of differentiation [31]. The overexpression of Pax7 in C2C12 myoblasts increased proliferation as expected, and delayed the onset of differentiation [32]. Contrarily, Pax7-null mice completely lacked satellite cells and were significantly smaller than their wild-type counterparts [33]. MSTN inhibits PAX7 activity through extracellular signal-related kinases (ERK) 1/2, and the antagonism of MSTN has been shown to increase PAX7 expression and populations of quiescent satellite cells [34]. MYOD commits cells of the dermomyotome to the myogenic lineage to form myoblasts [35,36]. In addition, MYOD initiates myoblast withdrawal from the cell cycle and differentiation to myocytes [37]. These cells proliferate and migrate to various locations where they fuse into multinucleated myotubes that further differentiate into functional muscle fibers [38]. Myogenin (MYOG) signals the completion of differentiation to myotubes and induces fusion into muscle fibers [39,40]. The MSTN signaling cascade results in the inhibition of the transcription of several MRFs, including MYOD and MYOG, to prevent the differentiation process into muscle fibers [40,41,42]. 

Overexpression of *MSTN-B* in particular skeletal muscles of transgenic quail impacted the expression pattern of PAX7, but the expression of other MRFs was unchanged compared to non-transgenic quail. This may be due to the different inhibitory mechanisms of MSTN on myogenic pathways. Both non-transgenic and transgenic embryos from A1 and A2 had expression of PAX7 at E13, which supports myogenic precursor cell proliferation. By E15, PAX7 expression decreased in non-transgenic embryos but remained significantly increased in transgenic embryos. The effects of this prolonged expression are unclear, but quail myogenic precursor cells overexpressing MSTN-B demonstrated increased cell density, which may be regulated by PAX7 [19]. Moreover, two bands appeared for PAX7 on the western blot; however, the band with a larger molecular weight was confirmed as the correct protein by overexpression of PAX7 in HeLa cells as the positive control. The band of smaller molecular weight could be an alternative splicing isoform that skipped exon 8 [43]. Although the expression pattern of PAX7 was different between MSTN-B transgenic and non-transgenic quail, the expression of MYOD and MYOG was not affected by the transgene, which may be because the amount of exogenous MSTN-B was not high enough to block the signaling cascade that inhibits the transcription of MRFs. 

In conclusion, transgenic quail lines overexpressing MSTN-B in particular skeletal muscles were generated. Within the gastrocnemius, exogenous MSTN-B had a pro-myogenic effect that increased the fiber number and prolonged PAX7 expression during embryonic development. Determining the amount of MSTN-B in the blood and ratios of MSTN-A to MSTN-B in muscle tissues would increase our knowledge about the role of MSTN-B in avians; however, a different promoter to drive the expression of MSTN-B in all skeletal muscle is required. MSTN-B contains the critical amino acid residues for binding and inhibiting MSTN processing; therefore, it may be a unique and useful selection marker for enhancing muscle mass in poultry species.

## 4. Materials and Methods 

### 4.1. Animal Subjects

Japanese quail (Coturnix coturnix japonica) used in this study were maintained at The Ohio State University poultry house (Lane Avenue) or at the Ohio Agricultural Research and Development Center (OARDC) turkey center as breeders. Quail were fed ad libitum on a standard starter or breeder diet. All animal procedures were approved by The Ohio State University Institutional Animal Care and Use Committee (IACUC; Protocol 2010AG0005-R2; Approved 2 February 2016). 

### 4.2. Vector Construction

The quail *MSTN-B* gene (qMSTN-B) with HA tag was amplified from pcDNA3.1-qMSTN-B-HA that was previously constructed in our lab by polymerase chain reaction (PCR) with forward primer including SalI site, 5′-TACTGTACGTCGACCTTCTGGTAACACATGCAA-3′, and reverse primer, 5′-TAGAAGGCACAGTCGAGG-3′, and cloned into the pCR2.1 cloning vector (Invitrogen, Carlsbad, CA, USA). The pLT-RSV-GFP lentiviral vector was cut with BamHI and SalI to remove the green fluorescent protein (GFP) coding region and ligated with qMSTN-B-HA cut from pCR2.1 with BamHI and XhoI. A 1.2-kb fragment of chicken skeletal muscle alpha actin 1 (cACTA1) was used to drive the expression of qMSTN-B in a skeletal muscle-specific manner [22]. Chicken skeletal muscle alpha actin 1 was amplified from genomic chicken DNA by PCR with forward primer including the ClaI site, 5′-AATCGATAGGGGATGCTGTGCTGTACC-3′, and reverse primer including the PacI site, 5′-AGTTAATTAATGTGCTGACTGCGCGTCG-3′, and cloned into the pCR2.1 cloning vector (Invitrogen). After cutting with ClaI and PacI, the cACTA1 promoter was inserted into pLT-RSV-qMSTN-B-HA cut with ClaI and PacI to remove the rous sarcoma virus (RSV) promoter. The final vector for generating MSTN-B transgenic quail is presented in Figure 1A.

### 4.3. Lentivirus Production and Concentration

Lentiviral particles were generated by calcium phosphate co-precipitation, as described by Shin et al. [44]. Briefly, 293FT cells were plated on 100-mm culture dishes with Dulbecco’s Modified Essential Medium (DMEM; Life Technologies Inc., Grand Island, NY, USA) supplemented with 10% fetal bovine serum (FBS; Life Technologies Inc.), 1% penicillin/streptomycin (pen/strep; Life Technologies Inc.), 0.1 mM of minima essential medium (MEM) non-essential amino-acids (Life Technologies Inc.), and 1 mM of sodium pyruvate (Life Technologies Inc.) until 95% confluent. The medium was changed two hours before transfection, and 9 μg of transfer vector (pLT-cACTA1-qMSTN-B-HA), 9 μg of ViraPower Packaging Mix (pLP1, pLP2, and pLP/VSVG; Life Technologies Inc.), and 87 μL of 2 M calcium solution (Clontech Laboratories Inc., Mountain View, CA, USA) were mixed to a final volume of 700 μL in sterile H_2_O (Clontech Laboratories Inc.). Then, 700 μL of 2× 4-(2-hydroxyethyl)-1-piperazineethanesulfonic acid (HEPES)-buffered saline (HBS; Clontech Laboratories Inc.) were added to both tubes by slowly vortexing. The DNA mixture was incubated at room temperature for 10 min and added dropwise to the complete medium in the plates. After 10 h, the medium was changed to 5 mL of fresh complete medium. Two days later, the medium containing the virus was harvested and filtered through 0.22-μm pore-size cellulose acetate filters. Lentiviral titers were measured by standard ELISA procedures using the Lenti-X p24 Rapid Titer Kit (Clontech Laboratories Inc.). The filtered medium was pelleted by ultracentrifugation (L7-65R, Beckman Coulter Inc., Brea, CA, USA) at 25,000 rpm for 2 h, resuspended in Opti-MEM (Life Technologies Inc.) to a concentration of 100×, and stored in 40-μL aliquots at −80 °C until use for injections.

### 4.4. Generation of Founder Quail

Fertilized eggs from wild-type quail were cleaned with 70% ethanol and placed laterally on a tray for 4 hours to orient the blastoderm to the lateral apex. Eggs were sanitized again, and a window 3 mm in diameter was cut out of the lateral apex of the eggshell using fine-tip tweezers. About 2 to 3 μL of concentrated lentivirus was injected into the subgerminal cavity of the blastoderm with a microinjection system (Tritech Research Inc., Los Angeles, CA, USA) under a stereomicroscope (Olympus America Inc., Center Valley, PA, USA). Then, the window was sealed with parafilm, and eggs were incubated for 14 days at 37.5 °C with 60% relative humidity until being placed in a hatching tray. The hatched chicks were grown to sexual maturity. 

### 4.5. Mating and Selection of Transgenic Offspring

Each mature founder was paired and mated with a wild-type quail. Eggs were collected daily, numbered by founder, and stored in a cold room (13 °C) until incubation. Hatched chicks were tagged and reared for two weeks to collect feather pulp for extracting genomic DNA. Briefly, feather pulp was incubated in 300 μL of cell lysis solution (CLS; 200 mM NaCl, 50 mM Tris-Cl, 10 mM EDTA, 1% SDS, pH 8.0) containing Proteinase K (1.5 μL/300 μL CLS, Invitrogen) at 55 °C for at least 3 h. To remove the protein, 300 μL of phenol–chloroform–isoamyl (Sigma-Aldrich, St. Louis, MO, USA) was added to the tube, vortexed, and centrifuged at 12,500× *g* for 2 min. The supernatant was transferred to a new tube, and 80 μL of 7.5 M ammonium acetate was added and centrifuged at 12,500× *g* for 10 min. From the supernatant, genomic DNA was precipitated by adding 240 μL of isopropanol, inverting, and centrifuging at 12,500× *g* for 5 min. The pellet was washed with 70% ethanol followed by drying and dissolving in TE buffer containing RNase A (10 mg/mL, Qiagen, Valencia, CA, USA). Genomic DNA was used for subsequent genotyping by PCR using the primer set, HA-tag-F: 5′-ACGACGTCCCAGACTACGCT-3′ for the forward primer and woodchuck hepatitis virus posttranscriptional regulatory element (WPRE)-R: 5′-AAGGGAGATCCGACTCGTCT-3′ for the reverse primer. Positive offspring were reconfirmed using the primer set, RRE-F: 5′-AATCGCAAAACCAGCAAGAAA-3′ for the forward primer and cACTA1-R: 5′-ATGAATGCTGGTGCCCTTAC-3′ for the reverse primer. The genotyping primer sets and amplicon lengths are described in Figure 1A. The confirmed G1 transgenic quail were used as breeders to produce G2 offspring for the study. 

### 4.6. Tissue Collection

Body weight was measured at hatch and then weekly until time of collection. From the selected lines that demonstrated strong expression of the transgene, five males and five females from non-transgenic and transgenic groups were euthanized by CO_2_ inhalation followed by cervical dislocation, and collections occurred at day 21 (D21) and D42. Males and females were collected separately to account for differences in growth between sexes. The pectoralis major (PM) muscle, right gastrocnemius (rGa), and heart (H) were excised and weighed. For normalization, the weights of the PM, rGa, and H were calculated as percentages of the body weight. A portion of the pectoralis major muscle and the right gastrocnemius were snap-frozen in liquid nitrogen and stored at −80 °C for further analyses. A cubical piece of pectoralis major that was positioned perpendicular to the fiber direction was removed from the upper left area of the muscle and was fixed for 24 h in Prefer fixative (Anatech Ltd., Battle Creek, MI, USA) before being stored in 70% ethanol at 4 °C. The left leg was cut through the femur, and the distal portion was fixed for 24 h in Prefer fixative (Anatech Ltd.). Then, the gastrocnemius was excised and placed in 70% ethanol at 4 °C until processing. Various tissues, including adipose tissue (A), pectoralis major (PM), thigh muscle (TM), heart (H), liver (Li), and lung (Lu) were collected from two non-transgenic and two transgenic quail from each line and stored at −80 °C to determine the tissue specificity of the transgene. Additionally, the pectoralis major muscle (PM), gastrocnemius (Ga), tibialis anterior (TA), triceps brachii (Tri), anterior latissimus dorsi (ALD), and posterior latissimus dorsi (PLD) were collected from two transgenic quail from each line and stored at −80 °C to compare transgene protein expression between different muscles. A total of 10 newly hatched chicks (D0) and 10 embryos at both embryonic day 13 (E13) and E15 were collected for each line. The entire right leg was excised and snap-frozen in liquid nitrogen for storage at −80 °C. 

### 4.7. Histological Processing

Pectoralis major muscle and gastrocnemius samples stored in 70% ethanol underwent sequential dehydration with 95% ethanol (2 × 60 min) and 100% ethanol (2 × 60 min); afterwards, they were cleared in xylene (2 × 45 min), and embedded in paraffin blocks. Slides were prepared with two or three serial 5-μm-thick slices using a microtome. Slides were deparaffinized in xylene (3 × 4 min). Ethanol hydration consisted of 100% (2 × 3 min), 95% (1 × 3 min), and 70% (1 × 3 min). Final hydration was performed in dH_2_O (1 × 3 min). Slides were placed in Harris hematoxylin (Thermo Fisher Scientific, Waltham, MA, USA) for 8 min and washed with running tap water for 8 min. Then, slides were dipped into acid ethanol (1% concentrated HCl in 95% ethanol) three times to remove residual hematoxylin. Slides were washed with dH_2_O (1 × 2 min), placed in Eosin Y (Thermo Fisher Scientific) for 2 min, dehydrated with 95% ethanol (2 × 2 min) and 100% ethanol (2 × 3 min), and cleared with xylene (3 × 3 min); afterwards, a permanent cover slip was mounted using Permount Mounting Medium (Thermo Fisher Scientific). Stained slides were observed and imaged with an AxioCam MRc 5 (Zeiss, Thornwood, NY, USA). ImageJ software (NIH ImageJ 1.47, http://imagej.nih.gov/ij) was used to determine the average fiber number in the gastrocnemius and average fiber cross-sectional area (CSA) in both pectoralis major muscle and gastrocnemius. The gastrocnemius was divided into four regions based on the total area. Counting was conducted by using ImageJ to determine the number of fibers across the entire median section of the gastrocnemius. For CSA, at least 500 fibers were evaluated per animal in both muscles. The average CSA was determined as the ratio of total area measured divided by total number of muscle fibers.

### 4.8. Total RNA Isolation and Quantitative Real-Time PCR

Total RNA from the gastrocnemius was isolated by using Trizol reagent (Invitrogen) according to the manufacturer’s protocol. RNA quality, cDNA synthesis, and qPCR was conducted as described by Choi et al. [45]. cDNA samples were used to conduct qPCR for myogenin (Myog). The primers were designed on different exons spanning genomic introns greater than 1 kb so as to prevent the amplification of genomic DNA, and the Myog primer set for qPCR was 5′-CTGCCCAAGGTGGAGATCCT-3′ for the forward primer and 5′-CTGGAGTTTGGCACCAACCC-3′ for the reverse primer. Conditions for qPCR were 95 °C for 10 min, and then 40 cycles of 94 °C for 15 s, 58 °C for 40 s, 72 °C for 30 s, and 82 °C for 32 s. ABI software was used to determine the target gene expression, and relative expression was calculated by the comparative 2^−ΔΔCt^ (CT: threshold cycle) method for relative quantification [46]. Ribosomal protein 13 (*RPS13*) was used as a housekeeping gene for normalization. 

### 4.9. Western Blot

Western blots were conducted as previously described by Choi et al. [45]. After blocking, membranes were incubated overnight at 4 °C with one of five primary antibodies: mouse hemagglutinin (HA) antibody (1:1000 dilution; Cell Signaling Technology, Danvers, MA, USA), PAX7 antibody (1:500 dilution; Developmental Studies Hybridoma Bank, Iowa City, IA, USA), MYOD antibody (1:1000 dilution; Santa Cruz Biotechnology Inc., Dallas, TX, USA), MSTN antibody (1:1000 dilution; AbClon, Seoul, Korea), or α-tubulin antibody (1:3000 dilution; Developmental Studies Hybridoma Bank) as a reference. The next day, membranes were washed six times in tris-buffered saline with Tween 20 (TBST) for 10 min each and incubated in either horseradish peroxidase-conjugated secondary anti-mouse immunoglobulin G (IgG) (1:5000 dilution; Jackson ImmunoReasearch Laboratories Inc., West Grove, PA, USA) or horseradish peroxidase-conjugated secondary anti-rabbit IgG (1:5000 dilution; Cell Signaling Technology) for 1 hour at room temperature. Membranes were washed six times for 10 min each followed by detection with ECL Plus (GE Healthcare, Piscataway, NJ, USA) and exposed on Amersham Hyperfilm ECL (GE Healthcare). Densitometry analysis was conducted by subtracting the background within the area measured for each band (Kodak 1-D Image Analysis Software, Eastman Kodak Company, Rochester, NY, USA).

### 4.10. Statistical Analysis

Densitometries and gene expression (2^−ΔΔCt^) for the quail lines were conducted by using one-way analysis of variance (ANOVA) within the general linear model (GLM) procedure of SAS 9.4 (SAS Inst. Inc., Cary, NC, USA) followed by Tukey’s honest significant difference test. Growth curves were analyzed by using repeated measures in the MIXED procedure. The Shapiro–Wilk test was used for evaluating the normality assumption for each experiment. Significance was discovered by testing hypotheses by using least square estimates. The type I error and family-wise error rate were controlled at a level of 0.05. Data were presented as least squares means and the standard errors of the means (SEM). 

## Figures and Tables

**Table 1:** Results of founder testcross for generating transgenic quail.

G0 Founder Quail		G1 Offspring		Line ID
Male	Female	Total Hatched	Transgenic (%) ^a^	
3234	Wild-type	44	5 (11.3)	A1
3240	Wild-type	56	1 (1.8)	A2
Wild-type	3242	46	4 (8.7)	A3

^a^ Percentage of G1 transgenic offspring among the total hatched chicks is designated in parentheses.

**Table 2:** Comparison of pectoralis major muscle, right gastrocnemius, and heart weights as percentages of body weight in non-transgenic (Non-Tg) and *MSTN-B* transgenic (Tg) male and female quail at D21 and D42 posthatch ^1,2^.

	D21 PMW/BW (%) ^3^	D21 rGaW/BW (%) ^4^	D21 HW/BW (%) ^5^	D42 PMW/BW (%)	D42 rGaW/BW (%)	D42 HW/BW (%)
**Non-Tg Male**	20.6 ± 0.43	0.365 ± 0.0041 ^a^	0.932 ± 0.026	22.4 ± 0.63	0.369 ± 0.0042 ^ab^	1.06 ± 0.047
**Tg** **Male**	21.4 ± 0.57	0.402 ± 0.011 ^b^	0.903 ± 0.030	23.2 ± 0.55	0.403 ± 0.0093 ^c^	1.08 ± 0.036
**Non-Tg Female**	21.4 ± 0.37	0.359 ± 0.0060 ^a^	0.922 ± 0.040	21.9 ± 0.73	0.344 ± 0.010 ^a^	1.01 ± 0.047
**Tg Female**	21.9 ± 0.61	0.394 ± 0.011 ^b^	0.962 ± 0.064	22.3 ± 0.54	0.403 ± 0.016 ^bc^	1.04 ± 0.27

^a–c^ Different superscripts within a column indicate statistical difference (*p* < 0.05); ^1^ Least squares means ± SEM; ^2^ *n* = 5 for all groups; Pectoralis major muscle weight as a percentage of body weight; ^4^ Right gastrocnemius weight as a percentage of body weight; ^5^ Heart weight as a percentage of body weight.

**Table 3:** Comparison of pectoralis major muscle (PM) cross-sectional area (CSA), left gastrocnemius (Ga) CSA, and left gastrocnemius fiber number in non-transgenic and MSTN-B transgenic male and female quail at D21 and D42 posthatch ^1,2^.

	D21 PM CSA (μm^2^)	D21 Ga CSA (μm^2^)	D21 Ga Fiber Number ^3^	D42 PM CSA (μm^2^)	D42 Ga CSA (μm^2^)	D42 Ga Fiber Number
**Non-Tg Male**	328 ± 27.6	909 ± 33.9	8182 ± 230 ^a^	498 ± 26.2	1191 ± 42.7	8394 ± 273 ^a^
**Tg** **Male**	362 ± 34.8	908 ± 36.9	9014 ± 214 ^b^	507 ± 32.6	1249 ± 43.8	9365 ± 231 ^b^
**Non-Tg Female**	371 ± 36.9	834 ± 31.3	8204 ± 266 ^a^	529 ± 27.9	1241 ± 47.3	8303 ± 237^a^
**Tg** **Female**	390 ± 32.5	851 ± 48.1	9188 ± 212 ^b^	502 ± 35.2	1270 ± 38.4	9345 ± 325 ^b^

^a,b^ Different superscripts within a column indicate statistical difference (*p* < 0.05); ^1^ Least squares means ± SEM; ^2^ *n* = 5 for all groups; ^3^ Total fiber number determined from a median section of the left gastrocnemius.

## Abbreviations

ACVR2B	activin receptor type 2B
ALD	anterior latissimus dorsi
BMP-1/TLD	bone morphogenetic protein-1/tolloid
cACTA1	chicken skeletal muscle alpha actin 1
CSA	fiber cross-sectional area
MRFs	myogenic regulatory factors
MSTN	myostatin
MYOD	myogenic differentiation 1
MYOG	myogenin
PAX7	paired box 7
PLD	posterior latissimus dorsi
PM	pectoralis major
rGa	right gastrocnemius
RPS13	ribosomal protein 13
TA	tibialis anterior
TGF-β	transforming growth factor-β
TM	thigh muscle
Tri	triceps brachii
